# Quality of Life in Prodromal HD: Qualitative Analyses of Discourse from Participants and Companions

**DOI:** 10.1155/2011/958439

**Published:** 2011-07-21

**Authors:** Rebecca E. Ready, Justin J. F. O'Rourke, Jane S. Paulsen

**Affiliations:** ^1^Department of Psychology, The University of Massachusetts, Tobin Hall, 135 Hicks Way, Amherst, MA 01003, USA; ^2^Department of Psychiatry, The University of Iowa, Iowa City, IA 52242, USA

## Abstract

Persons who are at risk for Huntington's Disease (HD) can be tested for the HD gene expansion before symptom onset. People with the gene expansion, but no clinical diagnosis, are in the prodromal phase of HD. This study explored quality of life (QOL) in prodromal HD. Interviews about QOL, conducted with 9 prodromal HD participants and 6 companions, were transcribed. Discourse was coded for emotional valence, content (e.g., coping, spirituality, interpersonal relationships, HD in others, and employment), and time frame (e.g., current, past, and future). Respondents were more positive than negative about the present, which was their major focus. The most common statements were about positive attitudes. Positive statements were made about spirituality, and negative statements were made about HD in other people. Relationships, employment, and coping with HD reflected both positivity and negativity. Participants and companions spoke of the future with different concerns. Applicability of findings to the clinical management of HD are discussed.

## 1. Introduction

Huntington's disease (HD) is a neurodegenerative disorder that causes a triad of cognitive, motor, and psychiatric symptoms [1]. It affects approximately 5–7 of every 100,000 persons worldwide [1]. It strikes persons in the prime of their lives, has an adverse impact on quality of life (QOL), and causes untold disability and suffering in patients and their loved ones [2–4]. Further, HD is the result of a defect in a single gene. If a parent has the HD gene expansion, there is a 50% chance that his/her biological children will inherit the disorder. The presence of the HD gene expansion indicates 100% certainty of developing HD at some point in life, if the individual does not die of another cause. 

The gene responsible for HD was identified in 1993; thus, persons can be tested to determine if they are *gene-expanded* (i.e., they have the HD gene expansion and will develop the disorder at some point in their lives) or *nonexpanded *(i.e., they do not have the HD gene expansion and they never will develop HD) [5]. It is this aspect of HD that makes it so exceedingly rare. Persons can know for certain if they will develop a neurodegenerative disease that, eventually, will affect control of their muscles, emotions, and thinking abilities, a progressive disease for which there is no cure and few effective treatments.

Persons who test positive for the HD gene expansion but have mild or no current symptoms are said to be in the* prodromal phase of HD* (in the past, persons who were gene-positive for HD but who did not meet diagnostic criteria for the disease were often said to be *presymptomatic *for HD; now, more often, persons in this stage of the disease are referred to as being in the *prodromal* phase of HD, in recognition that persons can suffer symptoms of HD prior to meeting full diagnostic criteria for the disorder). In the current project, nine adults with prodromal HD and six nonexpanded companions were interviewed about quality of life (QOL) in prodromal HD. Results provide insight into the psychological impact of knowing, in part, what the future holds. Currently, psychological research on genetic testing in prodromal HD focuses largely on decisions to undergo testing [6, 7] and reactions to test results [8, 9], but very little work has attended to QOL issues after testing is completed and a positive result is obtained. 

Reactions to test results, on average, suggest few long-term adverse consequences [8, 9], and some individuals benefit from test results by improving their interpersonal relationships, renewing their appreciation for life and exploring new directions (i.e., new careers) [10, 11]. Anxiety and depression temporarily increase for gene carriers, but, within a year, psychological symptoms return to baseline. Pessimism about the future and more suicidal ideation may occur [12, 13]. 

The current qualitative study adds to this line of work by focusing on a relatively unexplored psychological dimension of prodromal HD, namely, life quality. Measuring QOL is an important and relatively unexplored way to understand disease impact [14]. Given the lack of previous research on QOL in prodromal HD, the goal of the current study was to take an in-depth look at perceptions of QOL from a small convenience sample of persons who carry the gene expansion, as well as close companions. 

## 2. Methods

### 2.1. Participants

Participants self-reported that they had the gene expansion responsible for HD—confirmed through genetic testing—but were not currently diagnosed with the disease. Nine persons with prodromal HD (4 male; M age = 38.4 years, SD = 6.7; all Caucasian) participated. Each invited a spouse without the gene expansion or, in one case, fiancé to participate; six of the nine companions agreed to participate (3 male) (unfortunately, demographic information was not collected for the companions). Companions were only enrolled in the study if the participants with the HD gene expansion consented to their companions' participation and agreed to answer the same questions regarding themselves. All participants were recruited through a research registry or a monthly HD clinic at the University of Iowa Hospitals and Clinics Huntington's Disease Center of Excellence. 

All interviews were conducted individually and not in dyads. All participants provided informed consent, and the study was approved by the Internal Review Board at UIHC (200802793) and at the University of Massachusetts, Amherst (969), where data coding and analyses occurred; the study was conducted in accordance with the ethical standards of the 1964 Declaration of Helsinki. 

### 2.2. Procedure

Methods for data acquisition and coding were based largely on Hill and colleagues' Consensual Qualitative Research (CQR) approach, which is ideally suited for the early stages of research on previously unexplored topics [15]. Briefly, this method involves collection of data from small samples (e.g., *N*s = 8–15) via open-ended interview questions. Through an inductive and iterative process, content themes in the data are identified and coded; codes are verified by an auditor (uninvolved in the initial coding). Teams of researchers work on the project, and their multiple perspectives and differences of opinion stimulate rich exploration of the data. Individual interviews are coded, allowing for comparisons across participants.

In the current study, participants and companions were interviewed separately about QOL in prodromal HD. The interview questions were open ended (see the appendix) and were designed to prompt interviewees to think about QOL from a variety of different perspectives and to give maximal opportunity for persons to be able to reflect on their thoughts and ideas and life quality in an unstructured manner, without leading the participants to express certain ideas. All questions were asked of participants unless a participant spontaneously addressed an issue before being asked. Interviews were conducted by a trained, advanced graduate student (J. J. F. O'Rourke). The interviews lasted for 30–60 minutes and were conducted in person or over the telephone. Interviews were audio-recorded and transcribed verbatim (excluding identifying information) by trained research assistants (RAs). 

A single interviewer poses advantages and disadvantages. The advantage is that the interview is administered in roughly the same manner to all persons. A disadvantage is that idiosyncrasies or oversights that are particular to the interviewer could unduly influence the data. We selected one interviewer because he was an advanced graduate student in counseling and neuropsychology and had extensive experience with HD and prodromal HD in clinical and research contexts; another interviewer with the same expertise was not available. R. E. Ready reviewed all interviews to ensure quality.

#### 2.2.1. Identification of Thematic Content

All of the interviews were read by an initial team of three RAs, and, through an iterative process, common themes in the data were identified; the interviewer (J. J. F. O'Rourke) was not involved in this process to minimize bias in the analysis. Identification of prevalent content themes involved systematic thematic analysis by individuals, discussion in group meetings, and consensus meetings with R. E. Ready. Many meetings were held and content themes were refined (added, deleted, expanded, narrowed) as appropriate until a final list was agreed upon: coping (attitudes about life, approach to living, and behaviors, activities, and thoughts related to living and that impact mental or physical health), spirituality, interpersonal relationships, HD in other persons, employment, and other (e.g., personal symptoms of HD, which were rare, or vague/ambiguous statements that did not fit into a more specific category). 

#### 2.2.2. Coding

Transcripts were broken down into codeable units by the same three RAs. A codeable unit was defined as a complete thought and was usually a sentence; some complex sentences were broken down into more than one codeable unit. Each unit was coded along three dimensions: emotional valence (positive, negative, other (mixed/neutral)), thematic content (identified above), and time frame (present, past, future, other). 

Two independent RAs not previously involved in the identification of thematic content were trained on the coding system. Two interviews (one from a prodromal HD participant and one companion) were used for training purposes. The RAs coded them independently and then, together, reviewed ratings with R. E. Ready, and reconciled disagreements to improve interrater reliability when scoring the remaining interviews. Next, the remaining 13 interviews were independently coded by each RA, followed by group discussions with R. E. Ready, who served as the auditor, to reconcile discrepancies and achieve consensus; kappa agreement for each rating category was calculated prior to consensus meetings. 

### 2.3. Analyses

Analyses focused on frequency counts and cross-tabulations of statements with regard to emotional valence, themes, and time frame. Data from participants and companions were analyzed separately. Since some prodromal HD participants were part of a dyad (*n* = 6) and others were not (*n* = 3), results are presented for all gene-expanded participants (*n* = 9) as well as persons in dyads (*n* = 6). Separating out the participants in dyads facilitates comparison of participant and companion opinions about QOL. Selected excerpts from interviews illustrate the main findings. Lack of sum to 100% for results reported in tables and in the text reflects that some statements were coded as “other” (i.e., “other emotion,” “other time”).

## 3. Results

### 3.1. Interrater Reliability

Overall, coder agreement was fair but variable (Table 1). Stronger rater agreement occurred for easily identifiable content domains (i.e., employment, spirituality), emotional valence, and current behaviors/perceptions. Variability in coding was most notable for statements about the future, HD in other people, and coping. Statements about the future were occasionally vague and not always clearly linked to a time frame, which led to inconsistency when determining if statements were about the future or another time period. Coding statements about HD in other people was also variable due to potential overlap with the “interpersonal” category. To reduce overlap, statements that referred to manifestations of HD in others or witnessing HD in others were coded as “HD in other people,” whereas interpersonal interactions with persons affected by HD were coded as “interpersonal.” Agreement about statements related to coping were frequently ambiguous, but coding was improved by only including statements related to how persons were managing behaviors, thoughts, or feelings in relation to their (or their partner's) HD status.

It is a potential limitation that higher agreement could not be achieved for some categories; however, all final ratings were made in consensus conferences and thus discrepancies were resolved. Further, Hill et al.[15] cogently argue that in qualitative, exploratory research, differences in opinion are not necessarily a detriment to the process of discovery in the early stages of a line of research. 

### 3.2. Overall Frequencies

Statements about QOL were somewhat balanced between negativity and positivity (Table 1). The present was mentioned more frequently that the past or future. The most common content was related to interpersonal relationships and coping with HD status.

### 3.3. Emotion by Content Crosstabs

Examination of statements by emotion and content indicated that statements about employment were both positive and negative (Tables 2 and 3). For those in dyads, prodromal HD participants tended to be more positive about employment, whereas their companions exhibited more negativity. Prodromal HD participants and companions exhibited similar and fairly equal positivity and negativity when discussing interpersonal relationships. Coping tended to be more positive than negative for both groups.

Two content domains were highly valenced, meaning that they had stronger emotions associated with them than others. Spirituality was discussed in exclusively positive terms, even though it was the most infrequent content area. In contrast, HD in other people was more frequently discussed in negative terms.

### 3.4. Valence by Time Frame Crosstabs

Statements about the present were balanced somewhat more towards the positive than negative, whereas statements about the future and past were more often negative (Tables 4 and 5). It is perhaps not surprising that gene-expanded persons make negative statements about the future because it holds uncertainty related to the manifestation of HD and coping with the challenges of the disease. Negative statements about the past often referred to difficult decisions about undergoing gene testing, initial reactions to being gene positive, and interactions with family members around issues related to HD.

### 3.5. Time Frame by Content Domain and Three-Way Crosstabs

All content domains were well represented in present-oriented statements (Table 6). Similar to Table 2, spirituality and HD in others tended to be strongly positive and negative, respectively. For prodromal HD participants, comments about employment, interpersonal relationships, and coping were weighted more towards the positive (52%, 53%, and 63%, resp.) than the negative (25%, 34%, and 27%, resp.). Similar to prodromal HD participants, employment, interpersonal relationships, and coping were weighted more towards the positive (48%, 56%, and 61%, resp.) than the negative (12%, 14%, and 14%, resp.) for companions.

Future statements were not common, but, when they were made by gene-expanded individuals, they pertained most often to worries or concerns about maintaining employment when symptoms of HD begin to manifest. Furthermore, 91% of future statements by participants with prodromal HD about employment were negative and none were positive. When companions discussed the future, they most often talked about HD in other people and interpersonal relationships. For companions, more statements were negative than positive for the future of interpersonal relationships (63% versus 26%) and HD in others (80% versus 10%).

Statements about the past, while not common, tended to be in reference to interpersonal relationships, HD in others, and employment. For those with prodromal HD, past statements about HD in others were 67% negative and 3% positive; past statements about interpersonal relationships were 58% negative and 20% positive; past statements about employments were equally balanced positive (33%) and negative (33%). For companions, all these content domains tended to be more negative (56% for HD in others, 69% for interpersonal relationships, 80% for employment) than positive (0% for HD in others, 15% for interpersonal relationships, 20% employment). 

## 4. Discussion

Persons who are in the prodromal phase of HD and their companions are focused predominantly on the “here and now” rather than the past or the future. Thus, despite their unique knowledge about the future, they see QOL as being related to their present thoughts, activities, and behaviors. The most common statements were about the importance of a positive attitude. The importance of optimism came across in a variety of ways, but participants were clearly focused on living each moment to the fullest. Further, statements about spirituality, while rare, were uniformly positive. Helping persons with prodromal HD tap into or learn more about spirituality may be an effective means to increase life quality. 

With regard to the present, respondents were slightly more positive than negative. Relationships, employment, and efforts to cope with HD were discussed with a mix of positivity and negativity, suggesting that these factors can be assets and liabilities. The relative balance of positivity and negativity in these common life domains may be a hopeful sign for those in the prodromal phase of HD because it suggests potential to “tip the balance” of experiences in the positive direction; that is, if interpersonal relationships are associated with complex emotions but have positive attributes, it is theoretically possible to capitalize on the positive aspects while helping persons cope with the less adaptive aspects of their close relationships. In fact, efforts to capitalize on positive interpersonal perspectives have been discussed in the cancer literature, with many suggesting that interpersonal relationships can be actually *improved* beyond their precancer level, even while patients report concurrent interpersonal distress in their cancer experience [16, 17]. Similar efforts in prodromal HD populations may be particularly helpful when working to improve QOL. 

The most negative statements, not surprisingly, were about witnessing HD in other people, most often a family member. HD is a devastating disease that affects independence, movement, thinking, mood, and personality. Witnessing the manifestations of HD in a loved one is difficult, and observing the psychiatric symptoms of HD (e.g., depression, irritability, agitation, and aggression) can be particularly stressful [18]. When participants discussed negative reactions to observing HD in other persons, it was usually in reference to past experiences, which partially accounts for the negative valence surrounding statements about the past. 

These results suggest that interventions aimed at helping people in the prodromal phase of HD cope with their reactions to manifest HD in other persons may be particularly helpful. Providing instrumental and emotional support in coping with their past and current experiences with HD, in fact, might be one of the best ways to improve QOL since this issue was the biggest source of anxiety and distress in discourse about QOL. 

Although statements about past and present QOL were largely similar between companions and their gene-expanded partners, they differed in their concerns about the future. Persons with prodromal HD focused largely on occupational functioning, probably because threats to future employability are closely linked to income security and availability of health insurance. In contrast, companions' future concerns were about interpersonal relationships and HD in others. The companions' focus on these domains reflected concerns about the impact of progressing HD on close relationships, as well as their own ability to cope with uncertainty regarding their loved one's health. In summary, the worries of companions and prodromal HD individuals were different when looking to the future. These results suggest that family interventions aimed at preparing for future outcomes may be particularly helpful for improving QOL.

### 4.1. Sample Limitations

Participants in this study were unique. They pursued predictive genetic testing, whereas most persons at risk for HD choose *not* to undergo testing, indicating that participants in our sample may be more mentally resilient than those in the general HD population [8, 9]. Furthermore, many of them participate in research as a means of coping and to make a positive contribution to the future. Thus, they are motivated to know their own risk for HD and to advance research about the disease. For these reasons, results of this study may not generalize to other persons in the prodromal phase of HD. 

### 4.2. Conclusions and Future Directions

The current data suggest that QOL in prodromal HD might be enhanced by attending to spirituality, helping persons manage negative responses to HD in others, and by taking steps to maximize the positive and minimize the negative impacts of relationships, employment, and coping strategies. Along these lines, more knowledge about QOL in prodromal HD is critical to help affected persons, their families, and professionals learn what it is like for healthy persons to live with the knowledge that they are gene positive for HD. Attention to differential future concerns from persons with prodromal HD and their companions is warranted. 

Since genetic testing was made available in 1993, testing for HD has served as a model for other autosomal dominant disorders such as early-onset familial AD [19]. There is scant research on the psychological impact of genetic testing for familial AD, but one small study suggests that persons at risk for the disorder do not exhibit psychological symptoms as they approach age of onset [20]. The current study in prodromal HD sheds light on the importance of attending more deeply to psychological issues and life quality in persons at known or unknown risk for genetic disorders and can serve as a model for future work in familial AD and other autosomal dominant disorders. 

## Figures and Tables

**Table 1:** Percentage of codeable statements and kappa agreement for content, time, and emotion codes.

	All PrHD	PrHD in Dyad	Companions	Range	Mdn.
Valence					
Negative emotion	38%	36%	39%	0.35–0.92	0.68
Positive emotion	41%	43%	38%	0.11–0.90	0.71

Time					
Past	17%	17%	12%	0.38–0.76	0.52
Now	65%	66%	67%	0.00–0.84	0.72
Future	8%	7%	12%	0.20–0.67	0.48

Content					
Employment	6%	6%	6%	0.50–1.00	0.70
Spirituality	2%	1%	4%	0.33–1.00	0.87
Interpersonal relationships	31%	32%	24%	0.54–0.77	0.60
HD in other people	10%	11%	8%	0.09–0.67	0.40
Coping	27%	29%	37%	0.13–0.60	0.44

*N* = 15 (9 persons prodromal for HD; 6 companions).

**Table 2:** Valence of QOL statements by content domain.

	All PrHD		PrHD in Dyad		Companion	
	Positive	Negative	Positive	Negative	Positive	Negative
Employment	38%	12%	51%	26%	37%	53%
Spirituality	83%	0%	85%	0%	56%	20%
Interpersonal relationships	43%	40%	40%	46%	43%	42%
HD in other people	6%	72%	8%	74%	8%	60%
Coping	55%	32%	51%	34%	56%	30%

PrHD: prodromal for HD. All PrHD *N* = 9; PrHD in study dyad *N* = 6; companion *N* = 6.

**Table 3:** Sample positive and negative QOL statements by content domain.

Positive statements	PrHD	Companion
Employment	Well, one thing I did was change careers (to teaching).	Teaching… would be more of a positive experience.
Spirituality	My faith has become stronger because I need that to lean on.	She believes in God and lord as savior. She believes that one day everything will be better.
Interpersonal relationships	I know my husband will take care of me.	Well, she told me I'm a support system.
Coping	Quality of life is to live life to the fullest and experience most things that they can.	Good quality of life is finding satisfaction, I suppose, in the things that you do.

Negative statements	PrHD	Companion

Employment	I am not going to have a normal retirement age. I'm concerned; I want to make sure I am financially set.	He works nine hour days.
Interpersonal relationships	The emotional toll of being told (about being gene positive) and then the ramifications for both yourself and your family, is something that for me, at least, sent me into a tailspin for a while.	The other thing that really affects quality of life is if you have children, that was … probably the hardest part for my husband, which is the fact we had kids already and when we had children, he had no idea he was at risk.
HD in other people	I have seen some pretty negative things with this disease, as far as seeing people in my family who have HD.	It makes it hard to relate or communicate with my father-in-law because he is so negative or depressed.
Coping	I do worry about HD, I'll admit that.	We would both like to have more kids (but will not due to HD).

PrHD: Prodromal for HD. Sample statements within each row are paired, such that they are taken from a single dyad.

**Table 4:** Valence of QOL statements by time frame.

	All PrHD		PrHD in Dyad		Companion	
	Positive	Negative	Positive	Negative	Positive	Negative
Now	47%	35%	45%	38%	48%	31%
Future	27%	61%	27%	55%	31%	56%
Past	19%	53%	17%	51%	18%	61%

PrHD = Prodromal for HD. All PrHD *N* = 9; PrHD in study dyad *N* = 6; Companion *N* = 6.

**Table 5:** Sample positive and negative QOL statements by time frame.

Positive Statements	PrHD	Companion
Now	If you know (your gene status) you can start planning your life accordingly, living every moment.	Since you know that you have HD, you take that into consideration and you intentionally make decisions that are more impacting.
Future	I know it will be better for me than for my father because I know my husband will betaking care of me.	She knows that she is made secure because she is not going to be abandoned (in theFuture) because of this.
Past	My kids handled (results of gene testing) better than most adults.	We went on a trip to (foreign country) and it was totally awesome and I really feel like it was a great memory for my family.

Negative Statements	PrHD	Companion

Now	My kids and my husband; they are very distant. They don't want to hear about it or learnabout it.	I don't think I have helped much.
Future	The other thing that would make me sad would be for (my kids) not to have their mom.	It can be hard to know that eventually, and ultimately, you are going to get sick, so that would probably be one of the hardest things, just knowing.
Past	My grandchild was born and I showed no emotion.	Feeling guilty that maybe she passed on a gene to one of her kids and then one of her grandkids.

PrHD = Prodromal for HD. Sample statements within each row are paired, such that they are taken from a single dyad.

**Table 6:** Time frame of QOL statements by content domain.

	All PrHD			PrHD in Dyad			Companion		
	Now	Future	Past	Now	Future	Past	Now	Future	Past
Employment	58%	14%	19%	60%	13%	8%	66%	3%	26%
Spirituality	83%	4%	9%	92%	0%	8%	84%	8%	0%
Interpersonal relationships	67%	7%	21%	65%	7%	23%	66%	13%	18%
HD in other people	61%	3%	25%	59%	3%	24%	50%	19%	17%
Coping	75%	6%	10%	74%	6%	11%	77%	9%	9%

PrHD: Prodromal for HD. All PrHD *N* = 9; PrHD in study dyad *N* = 6; companion *N* = 6.

## Appendix

Potential interview questions that were used to stimulate discussion of quality of life (QOL) in presymptomatic Huntington's Disease (HD) Not all questions were asked to all persons or in the same way; they were adapted to each interview as it progressed.

1. *What does QOL mean for a person who is at risk for HD?*
  - *Is QOL different for persons who are not at risk for HD? What about compared to persons who already have the disease?*
  - *Is QOL different for a person who *knows *they are gene positive for HD versus a person who is at risk but has an unknown gene status? How? *
2. *What are some important determinants or predictors of QOL in persons at risk for HD? *
  - *What factors might increase QOL?*
  - *Decrease QOL?*
3. *How does knowledge about your future risk for HD impact your QOL right now?*
  - *Does worry about the future affect your QOL? How so?*
  - *What about feelings of guilt?*
  - *Does being secretive versus open about gene status affect QOL?*
  - *Do you have coping strategies that improve your QOL?*
4. *Are there any benefits to QOL in knowing your gene status? Any drawbacks? *
5. *Are there ways that QOL can be maximized for persons at risk for HD? How?*
6. *Are there certain persons, situations, or stressors that are particularly detrimental to QOL for persons at risk for HD?*
